# Application of a Conservative Prosthodontic Approach in the Rehabilitation of a 10-Year-Old Child with Hypohidrotic Ectodermal Dysplasia

**DOI:** 10.3390/healthcare13131543

**Published:** 2025-06-28

**Authors:** Abdulfatah Alazmah

**Affiliations:** Department of Pediatric Dentistry, College of Dentistry, Prince Sattam bin Abdulaziz University, Al-Kharj 11942, Saudi Arabia; a.alazmah@psau.edu.sa

**Keywords:** ectodermal dysplasia, conservative treatment, oral rehabilitation, dental anomalies, oligodontia

## Abstract

**Background/Objectives**: Hypohidrotic ectodermal dysplasia (HED) is a rare hereditary disorder affecting ectoderm-derived tissues including teeth, hair, and sweat glands. The dental abnormalities associated with HED, such as oligodontia and conical teeth, often result in significant functional, esthetic, and psychosocial challenges, particularly during childhood. **Methods**: A 10-year-old child presented with psychosocial concerns related to missing and malformed teeth. Clinical examination revealed oligodontia, conical anterior teeth, and a resorbed mandibular ridge. Based on clinical findings and a positive family history, a diagnosis of HED with significant dental involvement was confirmed. **Results**: A conservative prosthodontic approach was selected. A maxillary overdenture was fabricated over the retained primary teeth to enhance retention and preserve the alveolar bone, and a resin-bonded bridge was placed in the mandible due to poor ridge anatomy. The treatment restored oral function and esthetics and improved the child’s self-esteem. A recall visit after three months confirmed good prosthesis adaptation and a positive response from the patient and parents. **Conclusions**: This case highlights the importance of early, conservative, and developmentally appropriate prosthetic rehabilitation in pediatric patients with HED. Interim prostheses can significantly improve oral function, appearance, and psychosocial well-being while preserving future treatment options as the child matures.

## 1. Introduction

The Ectodermal Dysplasias (ED) Classification Working Group defines ectodermal dysplasias as a group of genetic disorders that impair the development and/or function of at least two ectodermal structures such as hair, teeth, nails, and certain glands. These conditions are genetically diverse and present a wide range of clinical features [1]. ED encompasses a heterogeneous group of over 200 distinct genetic disorders, of which hypohidrotic ectodermal dysplasia (HED) represents the most prevalent subtype, with an estimated incidence of 1 in 5000 to 10,000 live births and affecting 1 to 9 per 100,000 individuals [2]. It is predominantly associated with notable dental anomalies including hypodontia, anodontia, and conical or peg-shaped teeth. Craniofacial manifestations also often include frontal bossing, a saddle-shaped nose, reduced lower facial height, and a skeletal class III relationship. These abnormalities can severely impair oral function, disrupt speech development, compromise facial esthetics, and negatively influence the psychosocial development of affected children [3]. Therefore, early diagnosis of dental anomalies and prompt intervention are essential in the management of pediatric patients with ectodermal dysplasia, as functional and esthetic rehabilitation during childhood significantly enhances quality of life and psychosocial well-being [4].

Prosthetic rehabilitation is the main treatment of choice and is recommended in school-aged children to address nutrition, speech, and appearance. In cases of anodontia, complete dentures are typically the first line of treatment, with implant-supported options considered on a case-by-case basis from ages 5 to 10. Treatment planning for oligodontia and hypodontia may involve removable partial dentures or orthodontic management, often beginning around 6–10 years of age. Regardless of the approach, oral rehabilitation in children with ED should be patient-specific and minimally invasive [5].

Despite the availability of treatment guidelines and the recent classification of ED, the dental rehabilitation of patients with HED remains complex [6]. The rarity of the condition limits long-term clinical experience, making it difficult to predict outcomes with confidence. Consequently, treatment must be carefully individualized with a focus on flexibility, ongoing evaluation, and patient-centered care [7].

The management of ED in children is crucial to ensuring favorable long-term outcomes. Once the primary concern has been addressed, an interim treatment strategy should be implemented to support the longevity of the dentition and maintain tooth structure until definitive restorations can be provided, often several years later or until adulthood. Moreover, the child’s capacity to manage and adapt to dental treatment is a key factor influencing the choice and timing of restorative interventions, particularly in cases requiring complex or extended care [8].

This case report presents the dental rehabilitation of a pediatric patient diagnosed with ED characterized by oligodontia, highlighting the clinical challenges, treatment approach, and outcomes associated with managing this rare condition during the mixed dentition stage using a multidisciplinary conservative approach. Ethical approval was obtained by the Standing Committee of Bioethics Research (SCBR) at Prince Sattam bin Abdulaziz University (PSAU), with approval number SCBR-512/2025. Complete written informed consent was obtained from the patient and caregivers to use his data and images for publication.

## 2. Case Report

A 10-year-old healthy girl was referred to a Pediatric Dentistry Clinic for the diagnosis and treatment of multiple missing teeth and spacing, particularly in the mandibular anterior region. The esthetic impact of these missing teeth led to episodes of bullying at school, contributing to significant psychosocial distress as reported by her parents. Also, the existing teeth appeared smaller than average. A comprehensive dental and medical history was obtained from the patient and her parents. Family history revealed a pattern of dental anomalies: the patient’s mother had nine missing teeth, while her brother exhibited an agenesis of four teeth. Past dental history included regular follow-ups and one filling (upper right lateral incisor) without the need for local anesthesia. The referred dentist reported limited cooperation and dental anxiety during the visit. Intra-oral examination revealed a mixed dentition stage with both the primary and permanent dentitions free of carious lesions and normal soft tissue.

**Figure 1:**
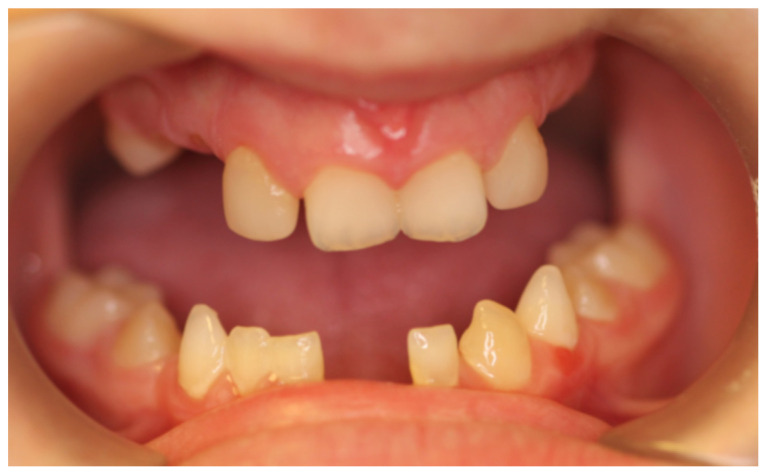
Intra-oral photograph, frontal view.

**Figure 2:**
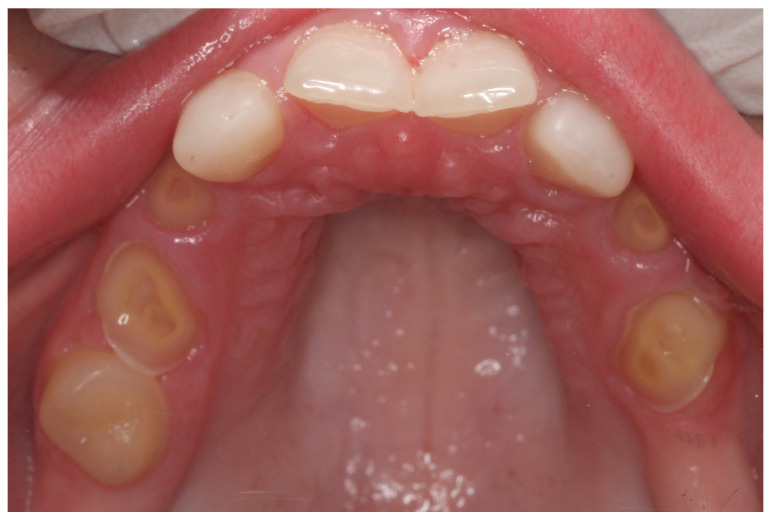
Intra-oral photograph of the upper arch.

**Figure 3:**
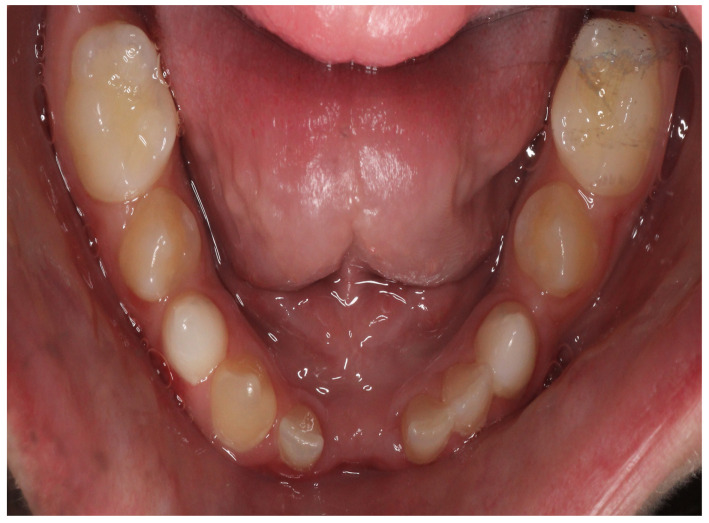
Intra-oral photograph of the lower arch.

At the initial visit, the permanent teeth present were 15, 34, 33, and 44. The primary teeth present were 75, 72, 81, 82, and 85. Several dental anomalies were observed: The mandibular primary canines (73 and 83) were conical in shape, the maxillary right lateral incisor (12) was peg-shaped, and microdontia was noted in the maxillary central and lateral incisors (11, 21, and 22). Erosive wear was also evident on the maxillary primary molars and canines (54, 53, 63, and 64), suggesting the structural weakening of enamel (Figure 1, Figure 2 and Figure 3).

**Figure 4:**
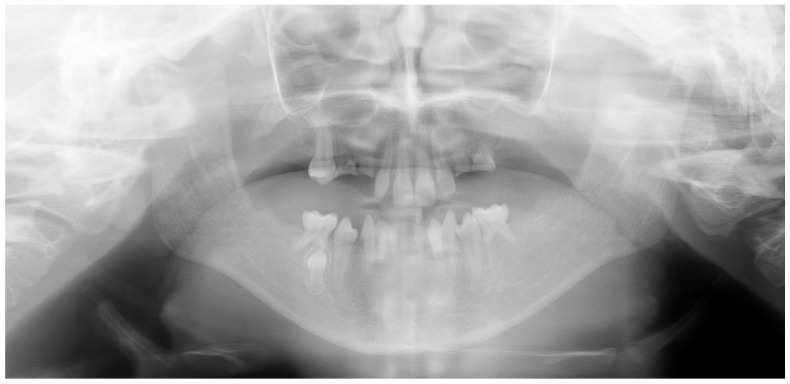
Dental panoramic tomogram (DPT).

The child exhibited a narrow maxillary arch along with skeletal class III, resulting in a complete lack of occlusal contact, along with a reduction in both overjet and overbite by approximately 2 mm. A midline assessment was not feasible due to the extent of dental anomalies. Reduced alveolar bone volume was observed to be associated with the underdevelopment of the alveolar ridge. The clinical findings were corroborated by a recent dental panoramic tomogram (DPT) provided by the referring dentist. Radiographic analysis confirmed the agenesis of nineteen permanent teeth, as well as the presence of an impacted mandibular right second premolar (tooth 45) (Figure 4).

After a thorough discussion with the patient and her parents and considering the child’s dental anxiety, the presence of multiple missing teeth, underdeveloped alveolar ridges, and reduced alveolar bone volume which presented a significant challenge for prosthetic rehabilitation, a conservative restorative approach was adopted to restore function and esthetics without compromising the already limited tooth structure. The non-invasive approach was selected to avoid the removal of tooth structure; hence, a non-pharmacological behavior management approach was used to improve the patient’s cooperation and reduce dental anxiety.

The treatment was structured into three phases: preventive, restorative, and maintenance. In the preventive phase, oral hygiene instructions were reinforced at every visit with a 22,600 ppm sodium fluoride varnish (Duraphat, Colgate, Palmolive, New York, NY, USA) applied weekly for three consecutive weeks to protect the existing dentition from further deterioration and prevent caries development [9].

During the restorative phase, treatment planning was approached through a multidisciplinary collaboration involving the Departments of Pediatric Dentistry, Orthodontics, and Prosthodontics [10]. Following consultation with the Orthodontics Department, it was recommended that orthodontic treatment be postponed until the completion of skeletal and dental growth. Removable prostheses were selected as an interim solution to improve mastication and appearance while accommodating future growth and potential definitive treatment options. The prostheses were carefully designed to ensure comfort, retention, and adaptability to ongoing dental development. The denture development stages were clearly explained to the child and the parents along with any expected challenges.

Preliminary impressions of both arches were taken using alginate (BluePrint X-creme, Dentsply International, York, PA, USA), and custom trays were fabricated with light-cured acrylic resin (Triad TruTray, Dentsply International, York, PA, USA). Final impressions were recorded using border molding with green stick compound (Impression compound, Kerr Corp, Brea, CA, USA), followed by a wash impression with medium-body vinyl polysiloxane (Aquasil Monophase, Dentsply Caulk, 78467, Kontanz, Germany).

For the maxillary overdenture, the non-infected primary teeth and their remaining roots were kept, preserving the space and the alveolar bone. The trial setup confirmed optimal esthetics, phonetics, and a balanced occlusal scheme, which were deemed satisfactory by both the patient and the parent, and retention was enhanced by incorporating an Adam’s clasp on tooth UR5 and a C clasp on tooth UL2. The treatment in the mandibular arch involved the extraction of the sound lower incisors teeth due to Grade II mobility, followed by the delivery of an immediate removable denture. Retentive clasps were placed on primary molars 75 and 85 to improve stability. The immediate denture was intended as a temporary solution to maintain function and esthetics during the healing period. Once the soft tissue had adequately healed, a conventional removable partial denture was fabricated to provide a more definitive prosthetic option.

**Figure 5:**
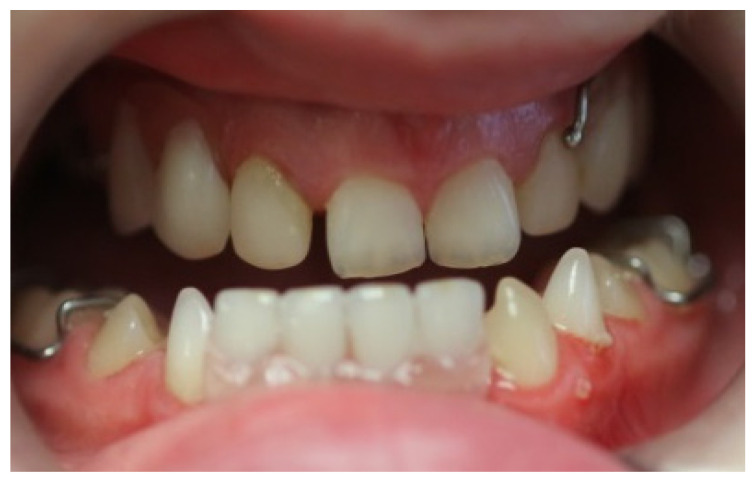
Intra-oral photograph after placement of upper overdenture and lower immediate denture.

Artificial teeth (VITAPAN, Vita, Zurich, Switzerland) were arranged and processed using heat-polymerized acrylic resin (Lucitone 199, Dentsply International, York, PA, USA). A laboratory remount procedure was performed to refine the occlusion before final polishing. The extraction of teeth 72, 81, and 82 was performed and hemostasis was achieved. At the insertion of the upper and lower dentures, pressure-indicating paste (PIP, Mizzy, Keystone Industries. GmbH, Germany) was used to assess fit with the necessary adjustments completed chairside (Figure 5).

The patient was reviewed 24 h and one-week post-insertion. Minor adjustments were performed at follow-up visits, and oral hygiene, as well as prosthesis care instructions, was reinforced. The patient was placed on a recall schedule for ongoing evaluation and maintenance.

**Figure 6:**
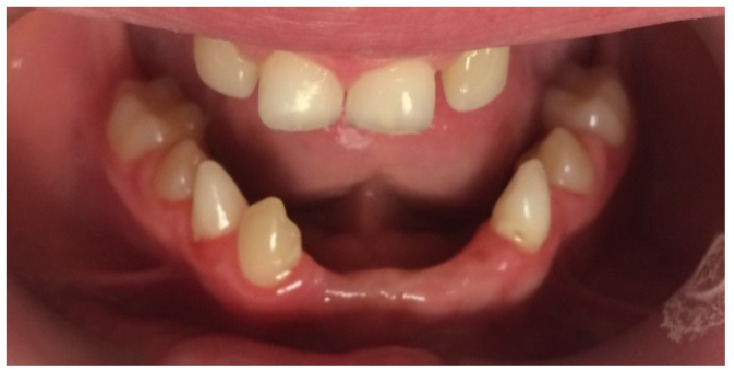
Intra-oral photograph, showing bone resorption after 1-month follow-up of immediate denture.

At the one-month review appointment, the patient reported poor retention of the lower immediate denture and wanted to discuss alternative solutions. Considering the significant alveolar ridge resorption in the lower jaw (Figure 6) and the child’s limited cooperation, a resin-bonded bridge (RBB) was selected as an alternative prosthetic solution to replace the missing mandibular anterior teeth. Teeth 33 and 83 were chosen as abutments based on their favorable periodontal condition and enamel surface. Shade selection was performed under natural light, and diagnostic records, including photographs and preliminary impressions, were obtained to aid in the wax-up and lab communication. Final impressions were taken with Silicon impressions using the light and heavy body, and bite registration was recorded with the opposing arch to ensure accurate articulation.

A metal-framed resin-bonded bridge was fabricated with pontics replacing the lower anterior teeth and retentive wings designed for bonding to the lingual surfaces of teeth 83 and 33. At the try-in appointment, the prosthesis was evaluated for marginal fit, shade accuracy, and occlusion, with special attention to ensure there were no occlusal interferences on the pontics. After confirming the fit, the bonding surfaces of the abutments were cleaned and etched with 37% phosphoric acid, rinsed, and gently air-dried. A bonding agent was applied to the enamel, and the wings of the RBB were cemented using a resin-based adhesive cement (Panavia V5, Kuraray Noritake, Okayama, Japan), with care taken to remove excess cement before light curing.

**Figure 7:**
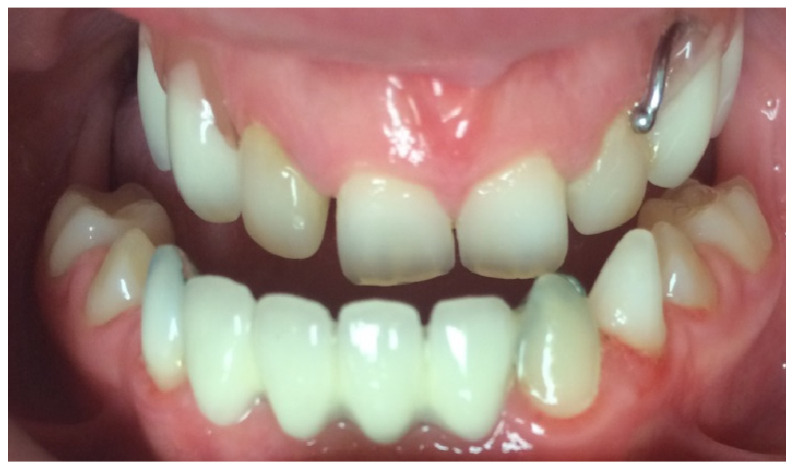
Intra-oral frontal view.

**Figure 8:**
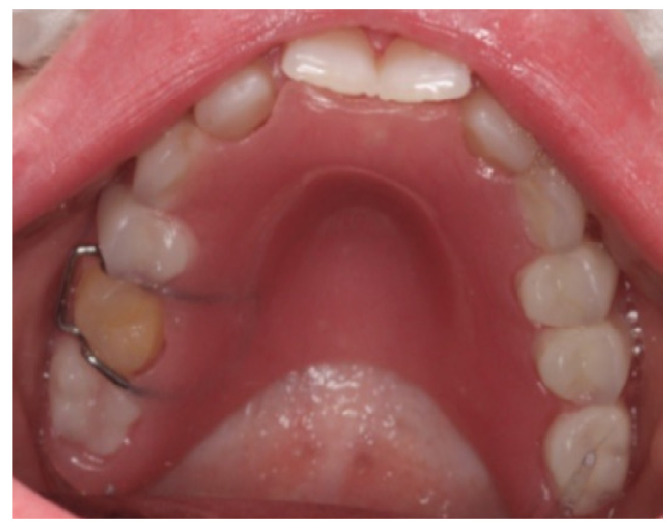
Intra-oral photograph, upper occlusal arch.

**Figure 9:**
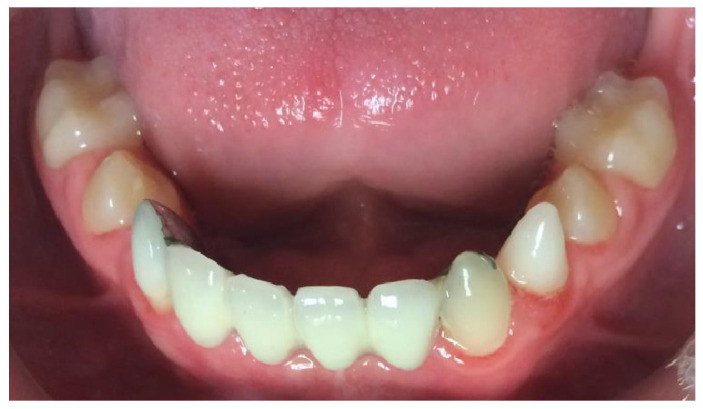
Intra-oral photograph, lower occlusal arch.

Post-insertion instructions included avoiding direct biting on the pontics for the initial 24 h and maintaining excellent oral hygiene, especially around the abutment teeth. The patient was reviewed one week, three months, and six months after cementation, with future follow-ups planned at regular intervals (Figure 7, Figure 8 and Figure 9).

## 3. Discussion

HED is a hereditary condition characterized by the abnormal development of ectodermal structures, notably affecting dentition. In the prosthodontic management of ectodermal dysplasia, treatment options may include fixed, removable, or implant-supported prostheses, used alone or in combination, to achieve optimal functional and esthetic rehabilitation [11].

In this case, a 10-year-old child with HED presented with classical oral manifestations including oligodontia, conical-shaped teeth, and skeletal class III. The average number of missing permanent teeth in patients with ectodermal dysplasia is reported to be approximately 23.7. In patients with ED, the most commonly missing permanent teeth are the maxillary central incisors (42%), maxillary first molars (41%), mandibular first molars (39%), and maxillary canines (22%) [12]. In our case, 19 permanent teeth were congenitally missing, and the pattern was consistent with typical findings. Notably, the maxillary central incisors and mandibular first premolars were present, while the maxillary canines and first molars, which are frequently absent in ED, were missing. This distribution reflects the characteristic hypodontia pattern associated with HED, which guided the prosthetic treatment planning accordingly. Although removable partial dentures are the most commonly chosen treatment, the final decision should involve a comprehensive discussion with the child and their parents. This should take into account the child’s preferences, treatment needs, tolerance for invasive procedures, and financial considerations [13]. The decision to use an overdenture in the maxilla was based on the availability of the existing tooth structure, which allowed for additional prosthetic support and improved retention. Overdentures are particularly advantageous in growing children, as they help preserve the alveolar bone and maintain vertical dimension more effectively than partial dentures. Van Waas et al. demonstrated that patients rehabilitated with overdentures experienced significantly less alveolar bone loss after two years compared to those with conventional dentures. Retention of primary teeth is often attributed to the developmental absence of their permanent successors, which is the most common underlying cause. This preservation of bone is critical in pediatric patients, as it contributes to long-term prosthetic stability and helps maintain facial esthetics during growth [14,15].

Similarly, fixed partial dentures (FPDs) provide a more stable and esthetically pleasing outcome, particularly when adequate abutment support is available. While Mehra and Grover noted that fixed partial dentures (FPDs) have historically been used less frequently in ED cases, they also highlighted a growing trend toward their use in children owing to their superior esthetics, improved retention, and greater functional stability in appropriately selected cases [6]. In our patient, a resin-bonded FPD was selected for the mandible due to compromised prosthesis retention. FPDs have been associated with improved occlusal vertical dimension and enhanced lip support, leading to superior esthetic and functional outcomes [16]. The mandibular arch was restored with a six-unit resin-bonded bridge supported by one permanent and one primary canine. The use of the lower FPD was supported by the favorable root anatomy of the canine abutments and the absence of an opposing occlusion, which minimized functional load, allowing for a stable and effective prosthetic solution. Following Ante’s law, the abutments provided sufficient periodontal support to replace the four missing incisors [17]. Additionally, since intercanine width is generally established between the ages of five and eight years, and the permanent canine in our patient had already erupted, minimal anterior transverse growth was anticipated [6,18].

Several case studies support this conservative and growth-sensitive approach. For instance, Parmar and Virdi (2025) reported successful prosthodontic rehabilitation of a nine-year-old child with HED using removable partial dentures, emphasizing the importance of non-invasive, adaptable treatments [19]. Compared to implant-based rehabilitation, which is often considered a long-term solution for patients with ED, the selected treatment strategy offers critical benefits in the pediatric population. Implant placement in growing children remains controversial due to ongoing craniofacial development, which may lead to infraocclusion, altered implant positioning, and the need for future revision surgeries. Furthermore, the reduced bone volume often seen in HED patients, particularly in the anterior mandible and maxilla, can complicate implant placement and compromise stability [20]. Our treatment approach also avoided subjecting the patient to early implant placement in the mandible. Although dental implants are commonly used in adult ED cases, studies involving pediatric patients remain limited, reflecting the rarity of the condition and the lack of robust clinical data. According to a recent systematic review, the number of implants placed in such cases varies from 2 to 12, with the most common configuration being 2 implants in the anterior mandible [21]. In our case, this option was further contraindicated by the child’s significant dental anxiety, which would have made surgical intervention and subsequent follow-up difficult. The distinctive use of a maxillary overdenture combined with a mandibular fixed partial denture (FPD) made this case unique. This prosthetic approach provided a minimally invasive, psychologically supportive, and functionally appropriate solution tailored to the patient’s developmental stage and emotional well-being.

The parents reported that the patient had significant improvement in speech and masticatory function following prosthetic rehabilitation. Beyond the physical benefits, early intervention also contributed positively to the child’s psychosocial well-being. Dental anomalies in children with ED often lead to social discomfort, low self-esteem, and vulnerability to teasing or bullying. By restoring oral function and improving facial esthetics, the treatment enhanced the child’s confidence and social interaction. This case highlights how timely prosthodontic care can play a pivotal role not only in oral health but also in supporting emotional and developmental outcomes in pediatric patients [3].

Replacement of full or overdentures is typically required every 1 to 3 years, while partial dentures may need to be renewed within 1 to 6 years, emphasizing the importance of regular follow-up and long-term maintenance planning in pediatric patients [7,22]. Follow-up care is essential to ensure prosthetic function and to accommodate growth-related changes. The patient was scheduled for regular follow-up during which prosthesis fit, oral hygiene, and functional adaptation were assessed. These periodic evaluations are critical to maintaining treatment success, monitoring tissue response, and planning for future interventions as the child matures, including orthodontic treatment [1,21].

Overall, the chosen treatment plan reflects a holistic, patient-centered approach that addresses both the clinical and emotional needs of pediatric patients with ectodermal dysplasia. Early intervention not only restores oral function and facial esthetics but also plays a vital role in enhancing quality of life and supporting healthy psychosocial development.

## 4. Conclusions

This case highlights the effectiveness of a conservative, growth-sensitive approach in managing a 10-year-old child with HED and 19 missing permanent teeth. A maxillary overdenture and mandibular resin-bonded bridge FPD were used to restore function, esthetics, and psychosocial well-being, while avoiding early implant placement due to the child’s age and dental anxiety. Future research should prioritize well-designed prospective studies with larger sample sizes and long-term follow-up to support evidence-based prosthetic planning in growing children.

## Data Availability

Data is contained within the article.

## Abbreviations

HED	Hypohidrotic ectodermal dysplasia
ED	Ectodermal Dysplasias
DPT	Dental panoramic tomogram
RBB	Resin-bonded bridge
FPD	Fixed Partial Denture
